# Chimpanzees Share Forbidden Fruit

**DOI:** 10.1371/journal.pone.0000886

**Published:** 2007-09-12

**Authors:** Kimberley J. Hockings, Tatyana Humle, James R. Anderson, Dora Biro, Claudia Sousa, Gaku Ohashi, Tetsuro Matsuzawa

**Affiliations:** 1 Department of Psychology, University of Stirling, Stirling, Scotland; 2 Department of Psychology, University of Wisconsin-Madison, Madison, Wisconsin, United States of America; 3 Department of Zoology, University of Oxford, Oxford, United Kingdom; 4 Department of Anthropology, New University of Lisbon, Lisbon, Portugal; 5 Primate Research Institute, Kyoto University, Kyoto, Japan; Georgia State University, United States of America

## Abstract

The sharing of wild plant foods is infrequent in chimpanzees, but in chimpanzee communities that engage in hunting, meat is frequently used as a ‘social tool’ for nurturing alliances and social bonds. Here we report the only recorded example of regular sharing of plant foods by unrelated, non-provisioned wild chimpanzees, and the contexts in which these sharing behaviours occur. From direct observations, adult chimpanzees at Bossou (Republic of Guinea, West Africa) very rarely transferred wild plant foods. In contrast, they shared cultivated plant foods much more frequently (58 out of 59 food sharing events). Sharing primarily consists of adult males allowing reproductively cycling females to take food that they possess. We propose that hypotheses focussing on ‘food-for-sex and -grooming’ and ‘showing-off’ strategies plausibly account for observed sharing behaviours. A changing human-dominated landscape presents chimpanzees with fresh challenges, and our observations suggest that crop-raiding provides adult male chimpanzees at Bossou with highly desirable food commodities that may be traded for other currencies.

## Introduction

Food sharing has important implications for the evolution of cooperation, offering a means to evaluate the ‘paradox’ of altruism, whereby a recipient gains fitness benefits at the expense of a donor [1]. When individuals control a highly valued resource, they may opt to use that resource as a tool for social bargaining. Thus, even acts that appear altruistic may serve to ultimately enhance one's own fitness.

Food sharing is observed throughout the animal kingdom, albeit at varying levels and complexities. Hypotheses proposed to explain food sharing behaviours in chimpanzees [for reviews], [see 1,2] range from cognitively simple explanations, such as begging intensity [3], to more complex sharing strategies, such as reciprocity [4]. Within chimpanzee communities that engage in hunting, meat is reportedly used as a ‘social tool’ [5]; alliances and affiliative relationships are cemented by gifts of meat. Long-term data from Mahale in Tanzania suggest that alpha males use meat sharing as a coalition strategy, never sharing with potential rivals such as beta or younger adult males [6]. However in Taï, Ivory Coast, hunters receive a share of meat if they participated in the hunt, regardless of the identity of the possessor [7]. The ‘meat-for-sex’ hypothesis suggests that males share meat with females either to gain immediate access to swollen females [8], [9] or to establish or strengthen an affiliative relationship and thus increase future mating opportunities [10]. Additionally, as meat is typically energetically costly and risky to acquire for chimpanzees, sharing with others may advertise an individual's strength and prowess [11]; simply possessing a desirable item may draw positive attention to an individual, enhancing the latter's social status [12], [13].

Analysis of such social and political components of meat sharing in chimpanzees has led to insights into the evolutionary basis of human food sharing [14]. Even though the major part of chimpanzees' diets consists of plant foods, wild plant food sharing occurs infrequently [15]–[18]. Following preliminary analyses [19], we report the regular sharing of raided plant crops between unrelated, non-provisioned wild chimpanzees at Bossou, Republic of Guinea, and describe the contexts of sharing behaviours.

## Results

Chimpanzee adults at Bossou very rarely transferred wild plant food (1 out of 59 food sharing events; excludes transfers of wild foods from mother to infant). This incident concerned the transfer of fig leaves *(Ficus exasperata)* from an adult male to his mother. Rough self-scratching (RSS), a likely behavioural indicator of anxiety [20], [21], especially in adult males [see K J Hockings 2007, DPhil thesis, Stirling University], was over 4 times higher when adult male chimpanzees acquired and fed on cultivated food compared to wild food (RSS rates, calculated from RSS frequency/focal observation time engaged in each feeding behaviour; *crop-raid*: 1.16 bouts/hr, *wild food*: 0.25 bouts/hr). All three adult males showed increased RSS (*alpha male*: 0.19^wild^ vs. 1.08^raid^ bouts/hr; *second-ranking male*: 0.44^ wild^ vs. 1.73^ raid^ bouts/hr; *third-ranking male*: 0.11^ wild^ vs. 0.55^ raid^ bouts/hr). Despite this, cultivated plant foods obtained during crop-raids were shared much more frequently than wild plant foods. Crop raiding bouts occurred on average 22 times per month (range: 5–45 bouts/month; SD±10.52), but crop sharing was much less frequent, occurring on average 1.5 times per month (range: 0–7 bouts/month; SD±2.43). This is in contrast to reports of hunting at other chimpanzee sites (*Gombe, Tanzania*: estimated at 8 hunts/months; *Taï*: 10 hunts/month; *Kibale, Uganda*: 4 hunts/month), most of which are followed by sharing [22]–[24]. Papaya, the largest and most easily divisible cultivated fruit available, was the most frequently shared crop type (36 out of 58 instances of sharing; binomial (0.165), p<0.001). Other cultivated plant foods were shared less often than papaya fruit (Table 1).

**Table 1:** Shows the frequencies (and percentages) of crop-raiding and crop sharing by the chimpanzees of Bossou.

Crop common name	Scientific name	Food part	Crop-raid annual events (% of annual total)	Crop share events (% of total events)
**Papaya**	***Carica papaya***	FT	130 (16.5%)	36 (62.1%)
		LF	69 (8.8%)	3 (5.2%)
		WT	0 (0%)	4 (6.9%)
**Banana**	***Musa sinensis***	FT	65 (8.3%)	0 (0%)
		PI	63 (8.0%)	0 (0%)
**Orange**	***Citrus aurantifolia***	FT	86 (10.9%)	5 (8.6%)
**Mandarin**	***Citrus reticulata***	FT	18 (2.3%)	0 (0%)
**Pineapple**	***Ananasa comosus***	FT	21 (2.7%)	3 (5.2%)
**Rice**	***Oryza sp.***	PI	81 (10.3%)	0 (0%)
**Maize**	***Zea mays***	FT	48 (6.1%)	1 (1.7%)
**Cassava**	***Manihot esculenta***	TB	74 (9.4%)	3 (5.2%)
**Cacao**	***Theobroma cacao***	FT	34 (4.3%)	2 (3.4%)
**Oil-palm**	***Elaeis guineensis***	FT	58 (7.4%)	0 (0%)
		NT	8 (1.0%)	0 (0%)
		FL	1 (0.1%)	0 (0%)
		PI	0 (0%)	0 (0%)
**Okra**	***Hibiscus esculentus***	LF	18 (2.3%)	0 (0%)
		FL	1 (0.1%)	0 (0%)
**Raphia-palm**	***Raphia gracilis***	GM	10 (1.3%)	0 (0%)
**Sugarcane**	***Saccharum officinarum***	PI	1 (0.1%)	1 (1.7%)
**Yam**	***Dioscorea sp.***	TB	0 (0%)	0 (0%)

Crop-raid event frequencies for a 12 month period and crop sharing frequencies for the complete study period, for each crop and part (FT: fruit, LF: leaf, WT: woody tissue, PI: pith, TB: tuber, NT: nut, FL: flower, GM: gum). Numbers in brackets indicate the percentage of total crop-raid events, and percentage of crop share events. In total, 786 crop-raiding events were observed throughout the 12 months, and 58 crop sharing events were recorded throughout the study period. The chimpanzees feed on 17 species of cultivated food; however certain species (e.g. mango fruit) are only consumed from abandoned orchards or fields. As these areas are not guarded, acquiring this food is not classified as crop-raiding.

**Figure 1:**
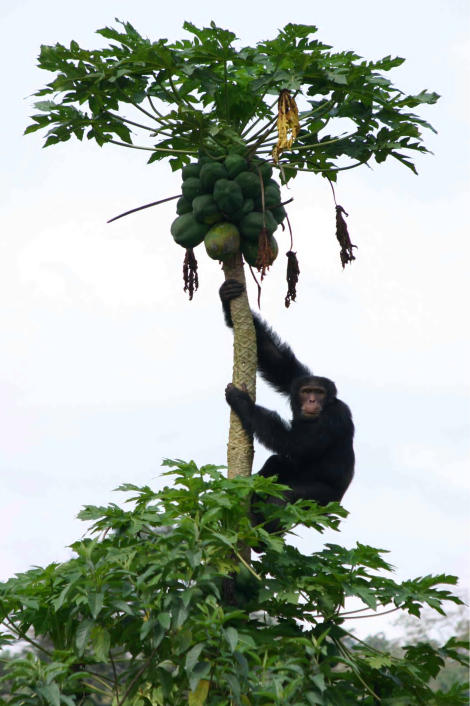
An adult male chimpanzee obtains cultivated papaya fruit.

Adult males were significantly more likely than other age- and sex-classes both to crop-raid and to share crops obtained in exposed locations (Figure 1; Movie S1 for supporting video), that is, in the village rather than the forest (*crop-raid*: *X* ^2^(1) = 21.835, p<0.001; *share*: *X* ^2^(1) = 9.54, p<0.01) and further from forest edge (*crop-raid*: Mann-Whitney U-test; Z = −3.711, p<0.001; *share*: Mean: 11 vs. 29 m, Z = −3.559, p<0.001). Adult males showed more RSS when raiding in the presence compared to the absence of local people (*X* ^2^(1) = 4.10, p<0.05), yet they were more likely than other age- and sex-classes to raid and share crops when local people were present (*crop-raid*: *X* ^2^(1) = 6.97, p<0.01; *share*: *X* ^2^(1) = 5.99, p<0.05). Males always transported raided foods from the village to the forest before sharing

**Figure 2:**
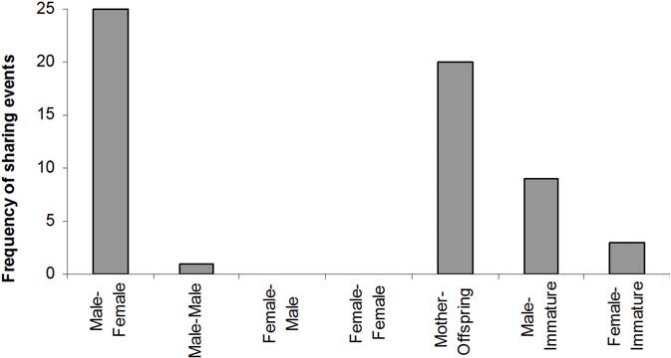
Age/sex classes of crop-sharing dyads. Offspring are of any age, ‘male’ or ‘female’ always refers to adults, and ‘immature’ refers to infants or juveniles less than 8-years old. Mother-offspring (includes offspring-mother) and male-immature sharing may be explained on the basis of kin selection.

Figure 2 summarises the crop sharing data. In over 33% of all papaya raids at least one male failed to obtain a fruit. However, males rarely shared with one another (only 1 out of 58 sharing events). Thus, crop sharing did not enhance cooperative raiding, as proposed for meat sharing at other sites. Females never shared crops with unrelated adults but 5 instances of crop sharing from offspring to mother were observed. The sharing offspring were adults in 4 out of these 5 sharing events (an adult female shared with her elderly mother on 1 occasion, and 2 adult males shared with their mothers on 3 occasions); there was one instance of a juvenile sharing with his mother.

Papaya raids occurred independently of the presence of females of reproductive age (*X* ^2^(1)  = 1.63, ns). However, adult males shared crops - mostly papaya (21 out of 25 sharing events) - overwhelmingly with these females (23 out of 25 sharing events with females; binomial (0.6), p = 0.0004), particularly with one cycling female (14 out of 23 events; Movie S2 for supporting video) who took part in 83% of all consortships with males. The second-ranking adult male, who shared most with this cycling female (43% of all her sharing episodes), was also her most frequent consort (50% of consortships) and grooming recipient (50% of her total grooming time). In comparison, the alpha male shared less frequently with this female (14% of all her sharing episodes), and despite his dominance, was less likely than the second-ranking male to consort (36% of consortships) and receive grooming (43% of her total grooming time) from her. Males shared crops with a maximally swollen female in 16% of sharing events, but were never observed mating with that female immediately after sharing. No aggressive interactions were observed during crop sharing episodes. In addition, clusters of individuals begging the possessor for a share of raided crops were rare; males shared mostly with a single female (19 out of 25 events; binomial (0.5), p = 0.005).

## Discussion

Cultivated plant foods are shared much more frequently than wild plant foods at Bossou, and are shared mostly by adult male chimpanzees. This seems paradoxical from the standpoint of the sharer, as adult males often appear nervous when raiding crops, it might be expected that they would be unlikely to share these costly foods. The shared cultivated fruits are usually large and easily divisible, and adult males are most likely to share such foods obtained in exposed locations and in the presence of local people (which is also associated with increased levels of arousal). As reported for meat sharing at other sites [11]–[Bibr pone.0000886-Teleki1] [13], crop-raiding adult males may be advertising prowess to other group-members; such daring behaviour may be considered an attractive trait [25], and possessing a desirable food item may further draw positive attention to an individual.

Our observations also raise the question of why adult male chimpanzees at Bossou regularly share raided fruits with females. Long-term data linking food sharing to male reproductive success would provide the most direct answer to this question, but in the absence of such data, we propose possible functions that appear consistent with the observations. Begging for crops appears to impose no energetic costs to the possessor; in contrast to begging for meat [3], aggression and the formation of begging clusters of individuals were absent. Furthermore, adult males shared especially with females of reproductive age, particularly with one cycling female who took part in most consortships. The adult male who shared most with this female engaged in more consortships with her and received more grooming from her than the other adult males, even the alpha male.

Meat sharing is infrequent at Bossou due to the scarcity of mammalian prey [26]; during this study it was only observed once: an adult female caught and shared a tree pangolin, *Manis tricuspis*, a species unlikely to inflict injury on its captor. Instead of meat sharing, the regular sharing of cultivated plant foods may be employed by adult males as a ‘food-for-sex and -grooming’ strategy to enhance affiliation with reproductively valuable females. Although the ‘food-for-sex and -grooming’ explanation can plausibly account for these observations, it relies principally on one cycling female, and may not be representative of the behaviour as a whole.

In humans, the pursuit of certain foods is also strongly sex-biased; it has been proposed that men in hunter-gatherer societies acquire large and risky-to-obtain food packages for social strategising and to garner attention [27]. Our observations suggest that crop-raiding provides adult male chimpanzees at Bossou with highly desirable and tradable food items. Additionally, individuals at Bossou sometimes share with their mothers. Although this can be explained by kin-selection, offspring-to-mother sharing is unreported in other wild and unprovisioned chimpanzee communities. As the unrelenting conversion of forested habitats to agricultural land continues; the potential for crop food sharing across chimpanzee populations seems likely to increase.

## Materials and Methods

Humans and chimpanzees coexist at Bossou (7 39′ N; 8 30′ W), where the 15km^2^ home range of the *Pan troglodytes verus* community is fragmented and surrounded by cultivated and abandoned orchards and farms. The chimpanzees regularly visit these areas to consume 17 varieties of crops that are located both within the village and forest. During the study period (February 2003 to December 2005, total of 454 study days), community size varied from 12 to 22 individuals; the number of adult females ranged from 4 to 8 individuals, and there was always the same 3 adult males [28]. All observed occurrences of crop-raiding, food sharing (defined as an individual holding a food item but allowing another individual to consume part of that item) and rough scratching, a self-directed behavioural pattern shown in response to anxiety, were recorded. During crop-raiding, the type and location of the crop, distance from forest edge (m) and local people's presence (auditory or visual) were noted. Females were classified as ‘of reproductive age’, ‘cycling’, or more specifically, ‘maximally swollen’. Consortships, where an adult female and an adult male move together to the periphery of their community range so that the male gains exclusive mating access, were also recorded.

## Supporting Information

Movie S1 An adult male chimpanzee crop-raiding papaya fruit. Adult male chimpanzees are most likely to share cultivated plant foods obtained in exposed locations and in the presence of people.
(10.26 MB AVI)

Movie S2 An adult male chimpanzee shares papaya fruit with an adult female. Cultivar food sharing at Bossou is the only recorded example of regular food sharing between unrelated and non-provisioned wild chimpanzees.
(8.76 MB AVI)
